# Adverse Childhood Experiences and Health Risk Behaviours among the Undergraduate Health Campus Students

**DOI:** 10.21315/mjms2023.30.1.13

**Published:** 2023-02-28

**Authors:** Mardhiah Majid, Azriani Ab Rahman, Fahisham Taib

**Affiliations:** 1 Department of Paediatrics, Hospital Universiti Sains Malaysia, Kelantan, Malaysia; 2 Department of Community Medicine, School of Medical Sciences, Universiti Sains Malaysia, Kelantan, Malaysia; 3 Department of Paediatrics, School of Medical Sciences, Universiti Sains Malaysia, Kelantan, Malaysia

**Keywords:** adverse childhood experiences, health risk behaviours, student, university, maltreatment

## Abstract

**Background:**

Adverse childhood experiences (ACEs) are linked with health risk behaviours (HRBs). The study aimed to evaluate ACEs in the undergraduate health campus of a public university located in the northeast of Malaysia and determine their association with HRBs.

**Methods:**

A cross-sectional study was performed by recruiting 973 undergraduate students at the health campus of a public university from December 2019 to June 2021. The World Health Organization (WHO) ACE-International Questionnaire and the Youth Risk Behaviour Surveillance System questionnaire were distributed using simple random sampling according to year of study and the selected batch of students. Descriptive statistics were used for demographic findings and logistic regression analyses were performed to determine the association between ACE and HRB.

**Results:**

The 973 participants (male [*n* = 245] and female [*n* = 728]) had a median age of 22 years old. The prevalence of child maltreatment among the study population was 30.2%, 29.2%, 28.7%, 9.1% and 6.1% for emotional abuse, emotional neglect, physical abuse, physical neglect and sexual abuse, respectively, among both sexes. The most commonly reported household dysfunctions were parental divorce/separation (5.5%). Community violence was 39.3% among the surveyed participants. The highest prevalence of HRBs among respondents was 54.5% from physical inactivity. The findings confirmed that those exposed to ACEs were at risk of HRBs and that a higher number of ACEs was associated with a higher number of HRBs.

**Conclusion:**

ACEs were highly prevalent among participating university students, ranging from 2.6%–39.3%. Hence, child maltreatment is an important public health problem in Malaysia.

## Introduction

Adverse childhood experiences (ACEs) are traumatic and stress-related events that occur during childhood before the age of 18 years old and the person remembers these events as an adult. Such events include multiple types of child abuse and neglect; exposure to violence between parents or household members; peer conflict and community violence; household dysfunction, including having an alcohol and/or substance abuser in the family, being single or having no parent, growing up with a mentally ill household member, or incarcerated household members (1).

ACEs can cause direct health consequences, such as somatic and psychosomatic disorders, cognitive-emotional reactions or even death (2, 3). Moreover, ACEs can also have a negative impact throughout the life course by affecting the individual’s physical and mental health (4, 5). Traumatic childhood experiences can be linked to various health risk behaviours (HRBs) later in life (6). Exposure to ACEs has been shown to be associated with HRBs, such as illicit drug use, sexual health risk and suicidal behaviour.

ACE study was first described in 1998 with the aim of exploring its prevalence. It was estimated that the prevalence of abuse among children around the world was as follows: 22.6% were physically abused, 36.3% were emotionally abused, 16.3% were physically neglected, 18.4% were emotionally neglected and 18% of girls experienced sexual abuse (7).

There were serious long-term effects of cumulative stress during childhood on physical, mental and HRBs among adolescents and adults who had ACEs. The risk of specific types of ACEs, such as violence and other adversities, varies considerably across different countries and cultures. However, the psychological and behavioural impacts are quite similar (8).

In Malaysia, there has been some research on HRBs in young adolescent groups. Unfortunately, the connection between ACEs and HRBs among young adults in Malaysia is presently unknown (9). This study investigates the prevalence and possible impacts of childhood adversities on HRBs among the undergraduates of a health campus at a local public university.

## Methods

### Methodology and Study Participants

A cross-sectional study was conducted by recruiting 973 Malaysian undergraduate students from three main faculties in the health campus of a public university: Medical Sciences, Dentistry and Health Sciences. Exchange and foreign students in this campus were excluded. Data were collected between December 2019 and June 2021. Midway to completion, the investigators faced a severe interruption in data collection due to the Movement Control Order (MCO) of the COVID-19 pandemic, imposed by the government of Malaysia.

### Sample Size and Sampling Method

The sample size was calculated using the single proportion formula. A study on ACEs and the Health of University Students in Eight Provinces of Vietnam was used to determine the proportion of university students with ACEs (10). Considering the 10% non-response rate and the design of the study, the minimum sample size required was 964.

Simple random sampling was used to select participants from the undergraduate students in each year of study and from different faculties. All eligible students from the selected batch were recruited.

### Tools and Data Collection

We utilised the World Health Organization (WHO) ACE-International Questionnaire and The Youth Risk Behaviour Surveillance System (YRBSS) year 2019 questionnaire. The WHO ACE-International Questionnaire has been validated in several languages (11). The questionnaire covers sociodemographic characteristics, household dysfunction, childhood maltreatment by parents or caregivers and witnessing community violence. It also has a forced choice yes/no answer to evaluate the experiences of participants before the age of 18 years old. Each ‘yes’ was scored 1 point, except for question 24 and 25, in which, the ‘no’ answer was scored 1 point. Therefore, the ACE score ranges from 0 to 11.

The English version of the YRBSS was used for HRBs. The YRBSS was developed in 1990 to monitor health behaviours that have contributed to the leading causes of death, disability, and social problems among youth and adults in the United States. A set of questionnaires was developed to suit the local setting (12). It measured six categories of priority health behaviours among youth that contribute to unintentional injuries and violence, sexual behaviours, alcohol, drug use, tobacco use and physical inactivity.

Data were collected either by administering a hard copy of the self-reported questionnaire or the online Google form questionnaire. This was done primarily because of social distancing and the implementation of MCO by the Malaysian government during the COVID-19 pandemic. Once ethical clearance was granted, the investigators enlisted a trained research assistant (RA) to carry out the fieldwork. Simple random sampling was used to determine the batch that would be approached, that is, medical, dentistry and allied health schools. Once the batch was selected, the RA briefed the selected group leader about the study. Further sampling was performed on the name list and selected students were invited for a voluntary participation in the study.

For the online participants, the study information was explained prior to the participants’ agreement. Only participants who chose ‘agree’ were able to proceed to complete the online questionnaire. Each participant was provided with information containing the helpline for psychological counselling in case there were issues related to the study. Each answered questionnaire was allocated a specific code to ensure confidentiality of the participants.

### Data Analysis

Data analyses were performed using IBM SPSS Statistics for Windows Version 24.0. Descriptive statistics were used for demographic data, proportion of ACEs, number of ACEs and HRBs. Simple and multiple logistic regression were used to analyse the association between ACE and HRB. All probability values were two-sided and a *P*-value < 0.05 was considered statistically significant. Basic assumptions were checked for logistic regression, including independence of errors, linearity in the continuous variables, absence of multicollinearity, and lack of influential outliers. The model fitness was assessed by goodness-of-fit measures.

## Results

### Sociodemographic Characteristics of Study Participants

Table 1 shows that the sample included in the study was formed by 973 participants, of whom 74.8% were female and 25.2% were male. The majority of study participants (95.5%) fell into the age group of 18 years old–24 years old. The mean age of participants was 22.38 years old.

Most of the students’ parents were employed (65.4% of mothers and 92.7% of fathers). These parents mostly have a college/university diploma or degree (48.6% of mothers and 51.1% of fathers).

### Proportion of Adverse Childhood Experiences among Undergraduate Health Campus Students

In Table 2, the highest forms of childhood maltreatment reported were emotional abuse (30.2%), emotional neglect (29.2%) and physical abuse (28.7%). Men had experienced emotional abuse (31% versus 29.9%), emotional neglect (32.2% versus 28.2%) and physical abuse (32.7% versus 27.3%) somewhat more frequently than women.

Emotional neglect in childhood was reported by 29.2% of participants. There were 18.9% of participants who believed that their parents did not understand their problems and 20.8% believed that their parents did not care about their children’s activities when they were not at school.

Physical abuse was experienced by 28.7% of participants in their first 18 years of life with one-fifth (21.8%) of participants stating their childhood experience of being spanked, slapped, kicked, punched or beaten. Physical neglect was experienced by 9.1% of participants, and 2.5% reported that they did not have enough food to eat during childhood.

A small percentage of participants reported exposure to sexual abuse in childhood (6.1% male and 46% female); 1.5% of men and 4.3% of women reported that in the first 18 years of their lives, at least one adult or someone five or more years older than them had touched or fondled their bodies in a sexual way.

A significant proportion of survey participants said they had witnessed community violence in real life. More than a third (39.3%) of participants had seen someone being beaten up, stabbed/shot in real life or threatened with a knife or gun.

### The Association between Different Types of Adverse Childhood Experiences

Table 3 shows that ACEs are often correlated with each other. Physical abuse often occurs with emotional abuse and sexual abuse and is experienced by those who have household members who are depressed or involved in crime/imprisoned. Sexual abuse (36.1%) was more often experienced by those who lived in households with someone who was depressed or had committed suicide.

Those who were exposed to household dysfunction reported that they had experienced physical abuse, emotional abuse and emotional neglect. These participants also reported to have had problems with community violence.

### Number of Adverse Childhood Experiences

Table 4 shows that 68.1% of the participants were positively marked for any of the 11 ACEs. The cumulative ACE score shows that 26% (*n* = 253) of the students had experienced at least one ACE; 15.6% (*n* = 152), two ACEs; 14.4% (*n* = 140), three ACEs; and 12.1% (*n* = 118), four or more ACEs. Male participants reported a higher number of ACEs than female participants.

### Proportion of Health Risk Behaviours

Table 5 shows the proportion of HRBs among participants. The HRBs include smoking, harmful alcohol and drug use, sexual risk behaviours, violence related behaviours, self-safety negligence and mental and health related issues.

The highest prevalence of HRBs among participants was 54.5% from physical inactivity, followed by being overweight/obese (28.8%) and safety negligence, including texting/emailing while driving (20.6%). Smoking prevalence was 12.4% in the whole group. Daily cigarette consumption was higher among male participants. However, surprisingly, the female participants reported a higher number of early smoking habits (before 8 years old).

Overall, the prevalence of alcohol use was 12.2% and that of illicit drug/marijuana use was 3.1%. Some participants (8.4%) reported having a known person in their family identified as an alcoholic. Male participants reported a significantly higher consumption of illicit drugs than female participants (7.8% of males versus 1.5% of females).

Approximately 5% of participants had sexual intercourse before the age of 18 years old. Men were more likely to have multiple sex partners than women; 44.4% of male participants with sexual risk behaviours had three or more sex partners. Of those who had sexual risk behaviours, 28.6% reported being sexually abused before age 18 years old.

Overall, 8% of participants reported having suicidal ideation in the past (7.8% of males vs 8.1% of females), and approximately 6.8% of them did attempt suicide.

Approximately one fifth (20.5%) of those with suicidal attempts reported feeling sad and hopeless almost every day for two weeks or more.

Half of the participants (54.5%) did not engage in enough physical activity according to the norms proposed by WHO. Female participants were reported to have lower physical activity than male participants (43.3% of males versus 58.2% of females). Approximately 28.8% of participants were overweight or obese.

### Association between different types of Adverse Childhood Experiences and Health Risk Behaviours

The relationships between different categories of ACEs and later involvement in different types of HRBs among the participants, was examined using regression analysis. If a participant was exposed to one ACE category, the probability of exposure to HRBs increased significantly. Participants who had been emotionally neglected during childhood were 2.26 times (AOR = 2.26; 95% CI = 1.040, 4.911) more likely to be involved in sexual intercourse. For those who lived with household members using street drugs, they were 2.40 times (AOR = 2.40; 95% CI = 1.141, 5.060) more likely to have physical inactivity.

### Association between Health Risk Behaviours and the number of Adverse Childhood Experiences Categories

The most significant relationship indicates that as a person is exposed to multiple ACE categories, the chances of becoming a smoker (AOR = 2.11; 95% CI = 1.275, 3.519), being involved in illicit drug use (AOR = 5.11; 95% CI = 1.391, 18.745), engaging in sexual intercourse (AOR = 4.35; 95% CI = 1.872, 10.147), feeling persistently sad and hopeless (AOR = 3.27; 95% CI = 1.923, 5.572), being bullied (AOR = 3.42; 95% CI = 1.642, 7.155) and having suicidal ideation (AOR = 3.62; 95% CI = 1.707, 7.687) increases significantly.

## Discussion

This is the first study performed locally to investigate ACEs and HRBs among the undergraduate students of a health campus. ACEs were highly prevalent among the participating students, ranging from 2.6% to 39.3%. At least 26% reported one form of ACE and 12.1% reported having experienced four or more categories of ACE, during the first 18 years of life. Emotional abuse and neglect, as well as physical abuse, were frequently reported forms of ACEs, which conformed with the findings of Ramiro et al. (13). Male students reported being more exposed to these types of ACE. This sex-related difference could be due to different perceptions of the parental insults, threats or negative emotions directed towards them. It is presumed that females are more prone to developing a higher tolerance than males. In general, a person who is emotionally and physically abused tends to naturally experience emotional distress (14).

In a previous study, a high percentage of participants was exposed to physical abuse before the age of 18 years old (27.5% of males and 26.6% of females) (15). Male participants reported frequently being pushed, grabbed, slapped (19.9% versus 18.4%) compared with female participants. Boys tend to be more often victims of physical abuse, similar to our study (32.7% of males and 27.3% of females) (16).

In our study, we documented a low exposure to sexual abuse (6.1%). The data on the prevalence of sexual abuse was often debatable because this form of maltreatment was quite difficult to talk about and narrate the experience compared to other forms of maltreatment. The 2012 ACE study in Romania estimated the prevalence of sexual abuse to be 10.9% for females and 5.6% for males (15). Stoltenborgh et al. suggested that the prevalence of childhood sexual abuse was 18% among females and 7.6% among males (7). These two studies suggest a higher prevalence of child sexual abuse among females than males. In our study, there was no difference in the prevalence of sexual abuse by sex (6.1% for males and 6% for females). This is probably due to the participants’ family background.

Exposure to physical neglect was reportedly low, similar to other developing countries. Parental neglect to basic needs may not be necessarily by intention, but rather due to concomitant social costs and lack of community support. Children may interpret material deprivation as a lack of parental care and love. Ney et al. (17) suggested that not having one’s biological needs met may make other types of maltreatment even more devastating. In our study, physical neglect in childhood was experienced by 11% of participants. Most of the participants stated concerns about food inadequacy similar to previous studies (15, 18).

We also documented household dysfunctions, such as having separated parents (5.5%). Divorce is often related to child maltreatment and poses a risk to the development and mental health of a child (19). The Department of Statistics Malaysia reported that the number of divorces in Malaysia increased from 50,862 (2018) to 56,975 (2019). Thus, the crude divorce rate increased from 1.6 (2018) to 1.8 (2019) per thousand population (20). Another prevalent household dysfunction was having a person with alcohol abuse in the family (4.3%). Students who reported living with alcoholics have a significant risk of becoming alcoholics themselves. Similar findings were seen in other studies (21).

The most prevalent HRBs were physical inactivity (54.5%), obesity (28.8%), self-safety negligence (20.6%), persistently feeling sad and hopeless (20.5%), smoking (12.4%) and alcohol use (12.2%). Males reported significantly more involvement in all risk behaviours. Another study found a weak association between physical inactivity, being overweight or obese and any category of ACEs (1).

There was a weak association between child maltreatment and other types of ACEs with depressive symptoms. This is different from other studies, which suggested that exposure to ACEs was associated with an increased risk of depressive symptoms up to decades after their occurrence (22).

Smoking is one of the most important risk factors associated with morbidity and mortality worldwide, which can be prevented. Twelve percent of the participants were active smokers. Males were reported to start smoking earlier than females. This sex difference was previously identified in the Health Behaviour in School-Aged Children 2010 survey (23). However, no increase in smoking practice was observed in those exposed to any type of ACEs.

The overall prevalence of alcohol use in our cohort was 12.2%. This rate is considerably higher than that in the current Malaysian setting. According to the Malaysian National Health and Morbidity Survey, the prevalence of alcohol consumption among adults aged 18 years old and above was 8.4% (24). Several studies have indicated that the use of harmful alcohol is related to childhood abuse or household dysfunction (25).

Male participants confessed that their first sexual intercourse was before 16 years old of age. This trend was similar to the survey results where 48% of 15-year-old boys reported that they had sexual intercourse at an early age (23). Our data also showed that males had more sexual partners in their lifetime than females. These results were similar to other studies which investigated sex differences in sexual behaviour. The analysis of the relationship between exposure to different ACE categories and HRBs showed that there was a 2-fold increase in sexual risk behaviours for those who experienced emotional neglect (AOR= 2.26; 95% CI = 1.040, 4.911).

Some participants in this study reported their involvement in physical fighting (3.9%) and that they had been bullied by their peers (4.1%). This finding was different from the European study where a larger number was documented (2). At least 39.3% of participants had witnessed community violence, with 37.6% of them reporting having witnessed someone being beaten up.

There is a relationship between the number of ACE exposures and HRBs. When the number of ACEs increased, there was also an increased risk of HRBs. These findings were in agreement with the previous studies, which found dose-response relationships between the number of ACEs and HRBs (2, 26). However, the associations in our study were unclear in selected HRBs, such as alcohol use, involvement in physical fights, and being overweight or obese. Other factors could potentially influence the outcome of such behavioural and health states, especially in adulthood.

## Conclusion

This study provides evidence that child maltreatment is a significant public health problem in Malaysia. Child advocates have important responsibilities to speak up for effective prevention and intervention strategies for child maltreatment. Understanding the crux of the problem, including the prevalence of ACEs and their relationship with HRBs is essential to reduce morbidity in later life. More research is required to investigate and improve screening methods, prevention programmes, and therapeutic interventions.

## Figures and Tables

**Table 1:** Sociodemographic characteristics of study participants

Characteristic		*n*	%
Individual age (in years old)		22.38 (1.43)	
		^*^Mean (SD)	
Gender	Male	245	25.2
	Female	728	74.8
Current marital status	Single	960	98.7
	Married	9	0.9
	Divorced	3	0.3
	Widow/Widower	1	0.1
Race	Malay	717	73.7
	Chinese	140	14.4
	Indian	85	8.7
	Others	31	3.2
Family type	Nuclear	854	87.8
	Extended	106	10.9
	Others	13	1.3
Mother’s level of education	Didn’t go to school	9	0.9
	Primary school	55	5.7
	Secondary school	436	44.8
	College/University	473	48.6
Father’s level of education	Did not go to school	8	0.8
	Primary school	70	7.2
	Secondary school	398	40.9
	College/University	497	51.1
Mother’s employment status	Employed	636	65.4
	Unemployed	315	32.4
	Retired	22	2.2
Father’s employment status	Employed	902	92.7
	Unemployed	23	2.4
	Retired	48	4.9

**Table 2:** Proportion of ACE among undergraduate Health Campus students

ACE category	Male *n* (%)	Female *n* (%)	Total *n* (%)
Physical abuse	80 (32.7)	199 (27.3)	279 (28.7)
Emotional abuse	76 (31)	218 (29.9)	294 (30.2)
Sexual abuse	15 (6.1)	44 (6)	59 (6.1)
Physical neglect	27 (11)	62 (8.5)	89 (9.1)
Emotional neglect	79 (32.2)	205 (28.2)	284 (29.2)
Separated or divorced parents	16 (6.5)	38 (5.2)	54 (5.5)
Depressed or suicidal by household member	6 (2.4)	30 (4.1)	36 (3.7)
Problem alcohol use by household member	11 (4.5)	31(4.3)	42 (4.3)
Street drug use by household member	4 (1.6)	28 (3.8)	32 (3.3)
Household member criminal or imprisoned	3 (1.2)	22 (3)	25 (2.6)
Community violence	124 (50.6)	258 (35.4)	382 (39.3)

**Table 3:** The association between different types of ACE among undergraduate Health Campus students

ACE category	*n* (%)	Physical abuse	Sexual abuse	Emotional abuse	Physical neglect	Emotional neglect	Community violence	Separated or divorced parents	Depressed or suicidal household member	Problem alcohol use by household member	Street drug use by household member	Household member involved in crime or imprisoned
Physical abuse	279 (28.7)		35 (59.3)	201 (68.4)	35 (39.3)	136 (47.9)	167 (43.7)	24 (44.4)	25 (69.4)	14 (33.3)	11 (34.4)	17 (68)
Sexual abuse	59 (6.1)	35 (12.5)		35 (11.9)	9 (10.1)	36 (12.7)	36 (9.4)	5 (9.3)	13 (36.1)	5 (11.9)	3 (9.4)	5 (20)
Emotional abuse	294 (30.2)	201 (72)	35 (59.3)		40 (44.9)	156 (54.9)	168 (44)	28 (51.9)	28 (77.8)	18 (42.9)	13 (40.6)	17 (68)
Physical neglect	89 (9.1)	35 (12.5)	9 (15.3)	40 (13.6)		42 (14.8)	43 (11.3)	9 (16.7)	5 (13.9)	7 (16.7)	3 (9.4)	3 (12)
Emotional neglect	284 (29.2)	136 (48.7)	36 (61)	156 (53.1)	42 (47.2)		139 (36.4)	20 (37)	25 (69.4)	18 (42.9)	15 (46.9)	18 (72)
Community violence	382 (39.3)	167 (59.9)	36 (61)	168 (57.1)	43 (48.3)	139 (48.9)		26 (48.1)	30 (83.3)	28 (66.7)	18 (56.3)	22 (88)
Separated or divorced parents	54 (5.5)	24 (8.6)	5 (8.5)	28 (9.5)	9 (10.1)	20 (7)	26 (6.8)		7 (19.4)	6 (14.3)	6 (18.8)	6 (24)
Depressed or suicidal household member	36 (3.7)	25 (9)	13 (22)	28 (9.5)	5 (5.6)	25 (8.8)	30 (7.9)	7 (13)		6 (14.3)	4 (12.5)	7 (28)
Problem alcohol use by household member	42 (4.3)	14 (5)	5 (8.5)	18 (6.1)	7 (7.9)	18 (6.3)	28 (7.3)	6 (11.1)	6 (16.7)		5 (15.6)	8 (32)
Street drug use by household member	32 (3.3)	11 (3.9)	3 (5.1)	13 (4.4)	3 (3.4)	15 (5.3)	18 (4.7)	6 (11.1)	4 (11.1)	5 (11.9)		13 (52)
Household member involved in crime or imprisoned	25 (2.6)	17 (6.1)	5 (8.5)	17 (5.8)	3 (3.4)	18 (6.3)	22 (9.8)	6 (11.1)	7 (19.4)	8 (19)	13 (40.6)	

**Table 4:** Number of ACE among undergraduate Health Campus students

Number of ACE	Male *n* (%)	Female *n* (%)	Total *n* (%)
0	56 (22.9)	254 (34.9)	310 (31.9)
1	69 (28.2)	184 (25.3)	253 (26)
2	45 (18.4)	107 (14.7)	152 (15.6)
3	43 (17.6)	97 (13.3)	140 (14.4)
4 or more	32 (13.1)	86 (11.8)	118 (12.1)

**Table 5:** Proportion of health-risk behaviours among undergraduate Health Campus students

Health risk behaviour	*n*	%
Cigarette smoker/electronic vapour use	121	12.4
Alcohol use	119	12.2
Marijuana/Illicit drugs use	30	3.1
Sexual intercourse	49	5
Feeling sad and hopeless almost every day for 2 weeks or more	199	20.5
Consider attempting suicide	78	8
Been bullied	40	4.1
Not wearing seat belt	10	1
Ride a car driven by someone who had been drinking alcohol	26	2.7
Driving drunk during past 30 days	9	0.9
Text or email during driving	200	20.6
Involve in physical fight	38	3.9
Overweight/obesity	280	28.8
Physical inactivity	530	54.5
